# No effect of dual exposure to sulfoxaflor and a trypanosome parasite on bumblebee olfactory learning

**DOI:** 10.1038/s41598-022-12714-3

**Published:** 2022-05-21

**Authors:** Owen P. Vaughan, Edward A. Straw, Alberto Linguadoca, Mark J. F. Brown

**Affiliations:** Department of Biological Sciences, Centre for Ecology, Evolution and Behaviour, School of Life Sciences and the Environment, Royal Holloway University of London, Egham, UK

**Keywords:** Animal behaviour, Entomology, Classical conditioning, Agroecology, Parasitology, Environmental impact, Ecology

## Abstract

Bees are important pollinators in wild and agricultural ecosystems, and understanding the factors driving their global declines is key to maintaining these pollination services. Learning, which has been a focus of previous ecotoxicological studies in bees, may play a key role in driving colony fitness. Here we move beyond the standard single-stressor approach to ask how multiple stressors, an agrochemical (sulfoxaflor, a relatively new insecticide) and a parasite (*Crithidia bombi*, a prevalent gut parasite of bumblebees), impact learning in the bumblebee *Bombus terrestris*. We developed a modified version of the classic proboscis extension reflex assay to assess the combined effects of acute oral sulfoxaflor exposure and infection by *C. bombi* on olfactory learning of bumblebee workers. We found no evidence that either sulfoxaflor, *C. bombi*, or their combination had any significant effect on bumblebee olfactory learning, despite their known negative impacts on other aspects of bumblebee health. This suggests that losses in cognitive ability, as measured here, are unlikely to explain the impacts of sulfoxaflor and its interactions with other stressors on bumblebees. Our novel methodology provides a model system within which to test interactive effects of other key stressors on bee health.

## Introduction

The importance of insect pollinators to ecosystems and agriculture cannot be overstated, with 87.5% of flowering plant species depending on or benefiting from their services^1^. Therefore, declines in the range, number and diversity of bees across many regions^2,3^ is of significant concern, especially as bees account for 62% of flowering crop visitations^4^. Bumblebees provide a major portion of these pollination services, both as wild and as commercially produced pollinators (e.g., *Bombus spp.* are the second most commercially used agricultural pollinators^5,6^ after the European honeybee *Apis mellifera*^7^)*.*

A multitude of stressors are driving bee declines, the most important of which are habitat loss and fragmentation, a loss of food resources, pressure from invasive species, the effects of climate change, pathogen and parasite infections and exposure to pesticides^8–11^. Due to the nature of modern agriculture, bees are exposed to a diverse range of pesticides^12–16^. Similarly, bees are hosts to numerous parasite species that may play a role in their decline^17–20^. Consequently, there is significant opportunity for dual exposure to pesticides and parasites. Partly as a result of this, the combined effects of stressors, such as the accumulation of sublethal factors caused by diseases and anthropogenic pressure, are gaining more attention^21–24^.

In social bees, like bumblebees and honeybees, a foraging bee’s ability to bring food resources to the colony relies heavily on its ability to learn and remember which flowers are best to forage from, where they are located relative to the colony, and what is the optimal route between one and the other^25,26^. Impairment of the cognitive abilities of the foragers of a colony could significantly impact its food supply, due to the variation in location and abundance of desirable flower resources^26–28^. Therefore, any stressors that are detrimental to the learning abilities of bee foragers, such as olfactory learning or navigation, may impact colony fitness and growth in a significant way^29^.

The Proboscis Extension Reflex protocol (PER) is a widely used paradigm to test learning in bees^30^ and has been in use for several decades^31,32^. Bees extend their proboscis to collect nectar from flowers, and this reflex can be exploited alongside classical conditioning to assess olfactory learning and memory^33^. A large proportion of the studies on the effects of pesticides and pathogens on bee learning and memory test only a single stressor at a time^30,34^. Due to its well-established nature for testing olfactory learning in bees, PER can be used to develop a multi-stressor study for pesticides and parasites on bumblebee learning. Here we develop a protocol and provide results for a PER-based study testing multiple stressors. We use *B. terrestris* as our study organism. While *B. terrestris* may not be under threat, it is a useful model organism representing other bumblebee species and is an important wild pollinator that has widespread commercial use^6,9,35,36^. In addition, the majority of PER studies for pesticide effects have focused on *A. mellifera*^30^, so studying an alternative species combats the dearth of studies in bees other than honeybees.

Sulfoxaflor is a sulfoximine insecticide, that has been approved for use in 81 countries^37^. Sulfoximines are systemic insecticides, meaning that they are taken up and distributed throughout plant tissues, and act as nicotinic acetylcholine receptor (nAChR) competitive modulators, which overstimulate the insect nervous system, resulting in paralysis and death^38^. Sulfoxaflor’s mode of action is similar to neonicotinoids, which are also systemic insecticides and nAChR competitive modulators^39,40^. Given that neonicotinoids are known to have negative effects on bee learning and foraging behaviour^29,30,41–45^, and their similar mode of action, sulfoximines might be expected to have similar effects. However, Siviter et al. (2019) found no evidence for a negative effect of acute exposure to sulfoxaflor on olfactory learning and memory in *A. mellifera* and *B. terrestris*^37^. It is not well understood why this may be, but sulfoxaflor is known to interact differently with nAChRs and metabolic enzymes in insects than neonicotinoids do^46^. Little is known about how sulfoxaflor might interact with bee parasites. Consequently, Siviter et al. (2019) results provide the baseline for a multi-stressor study, and so here we chose to acutely expose bumblebees to sulfoxaflor for our combined effects protocol.

The trypanosome gut parasite *Crithidia bombi* is a widespread and highly prevalent parasite of bumblebees^47–50^. This makes it an obvious parasite to use for the first multi-stressor PER study in bumblebees. In addition, *C. bombi* is known to impair visual learning and foraging activity in bumblebee workers^51,52^, although it has not been found to affect bumblebee olfactory learning^53^. The latter fact provides a baseline for validating and interpreting results from this multi-stressor study.

Although these two stressors are, on their own, not known to significantly affect olfactory learning in bumblebees, it does not mean they might not interact, resulting in significant effects, even if a possible mechanism behind such an interaction is unclear. Additionally, the dose of sulfoxaflor we used was much greater than those previously used^37^. It was unknown if such a dose of sulfoxaflor would significantly affect bumblebee olfactory learning on its own.

## Results

**Figure 1:**
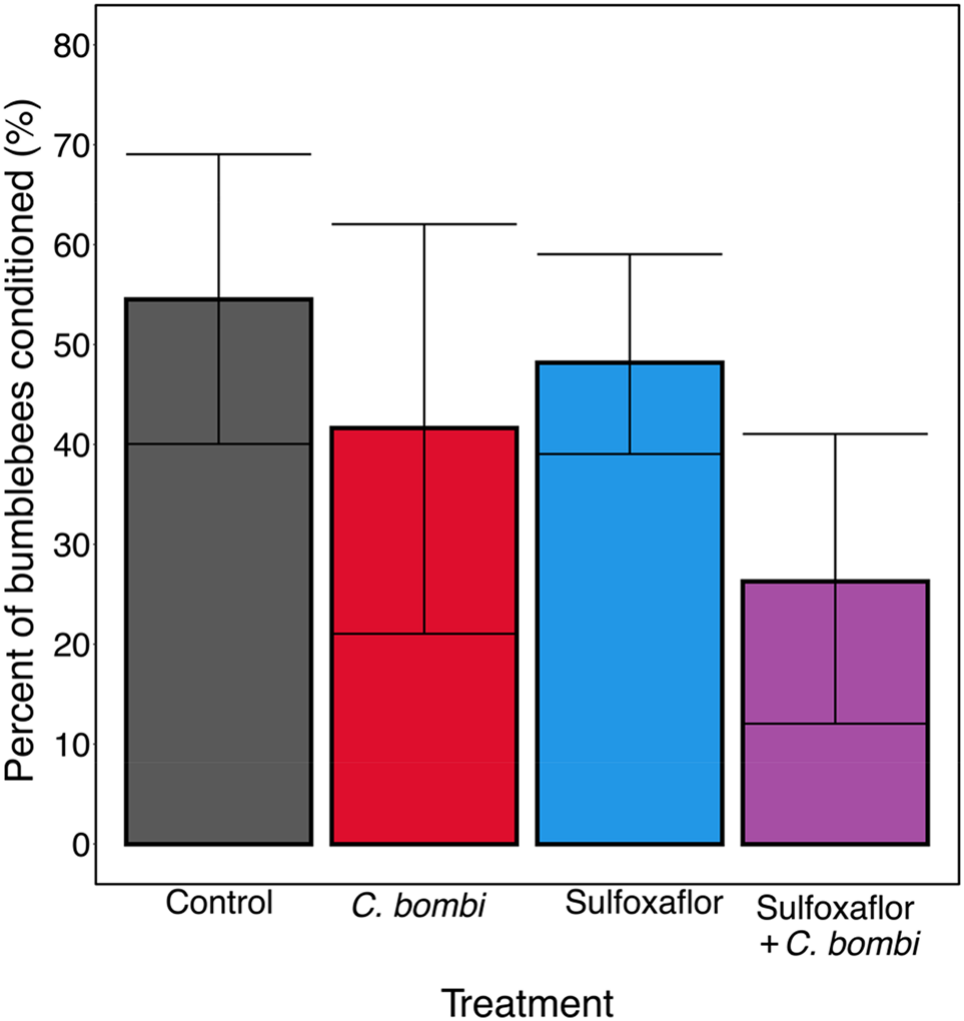
Responsiveness. The proportion of workers that showed a conditioned response in each treatment group ± 95% confidence interval (control n = 44; sulfoxaflor n = 56; *C. bombi* n = 24; sulfoxaflor + *C. bombi* n = 38).

For Responsiveness (whether or not a bee gave any conditioned responses), we found no evidence of significant impacts of exposure to sulfoxaflor, infection by *C. bombi* or their combination (Fig. 1; General Linear Model (GLM) Parameter Estimate (PE) =  −0.09, 95% CI [−0.62 to 0.43]; *C. bombi* PE =  −0.14, 95% CI [−0.84 to 0.56]; sulfoxaflor and *C. bombi* PE = -−0.29, 95% CI [−1.37 to 0.79]). Bees exposed to sulfoxaflor gave a conditioned response 48% of the time, those exposed to *C. bombi* 42% of the time, and to both sulfoxaflor and *C. bombi* 26% of the time, in comparison to the control at 55% of the time.

**Figure 2:**
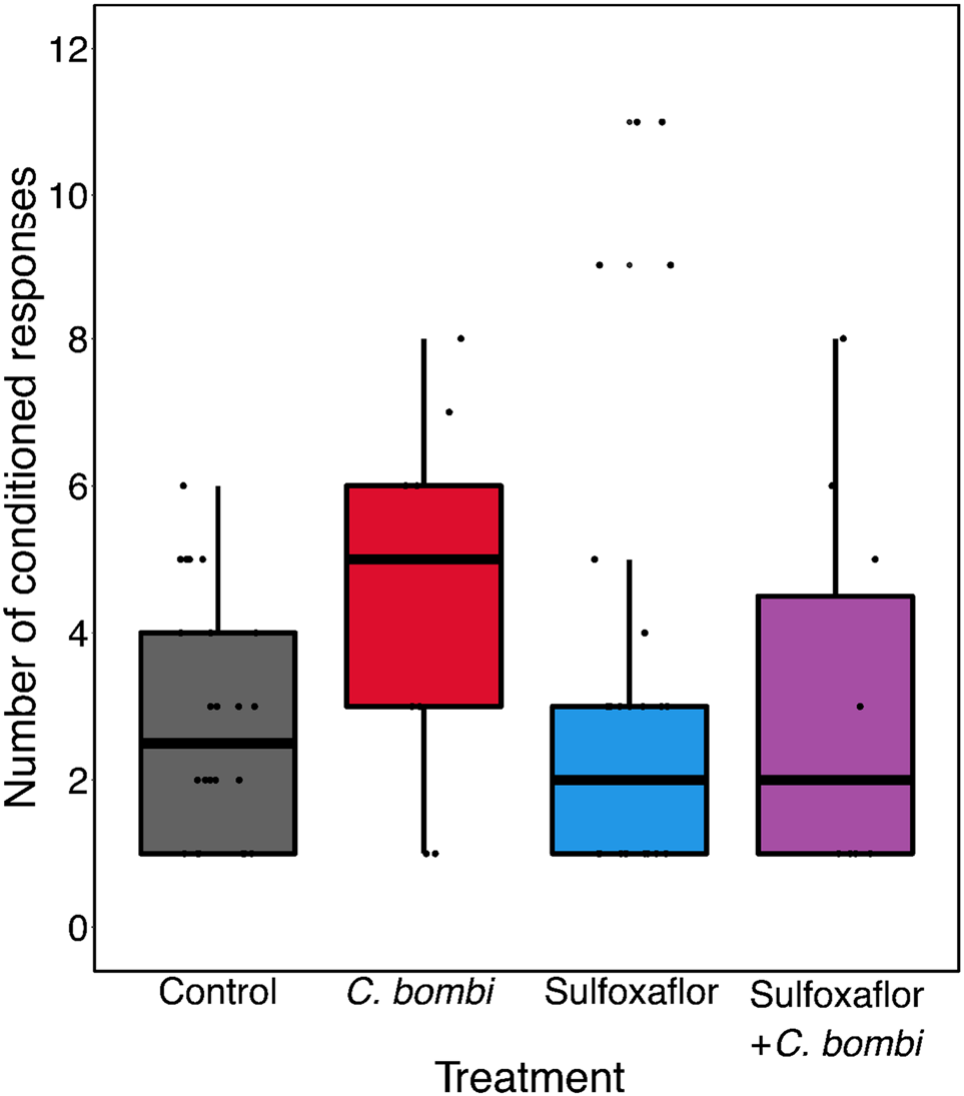
Learning Level. The number of conditioned responses responsive workers showed in each treatment group ± 95% confidence interval (control n = 24; sulfoxaflor n = 27; *C. bombi* n = 10; sulfoxaflor + *C. bombi* n = 10). The number of bees differs from the other figures as this analysis only took responsive bees into account.

For Learning Level (number of conditioned responses a bee gave), again we found no evidence of significant impacts of exposure to sulfoxaflor, infection by *C. bombi* or their combination (Fig. 2 Quasi-poisson model; results from least complex model containing the treatment variable are presented for comparability; sulfoxaflor Parameter Estimate (PE) = 0.15, 95% CI [−0.28 to 0.60]; *C. bombi* PE = 0.48, 95% CI [−0.06 to 1.00]; sulfoxaflor and *C. bombi* PE = 0.07, 95% CI [−0.55 to 0.65]), as the null-model (not containing the treatment term) was retained as the standing ‘best model’ (see Supplementary Fig. 2 for comparison statistics). Bees that had conditioned responses and were exposed to sulfoxaflor showed on average 3 conditioned responses, those exposed to *C. bombi* showed 5, those exposed to both sulfoxaflor and *C. bombi* showed 3, in comparison to the control which showed 3.

**Figure 3:**
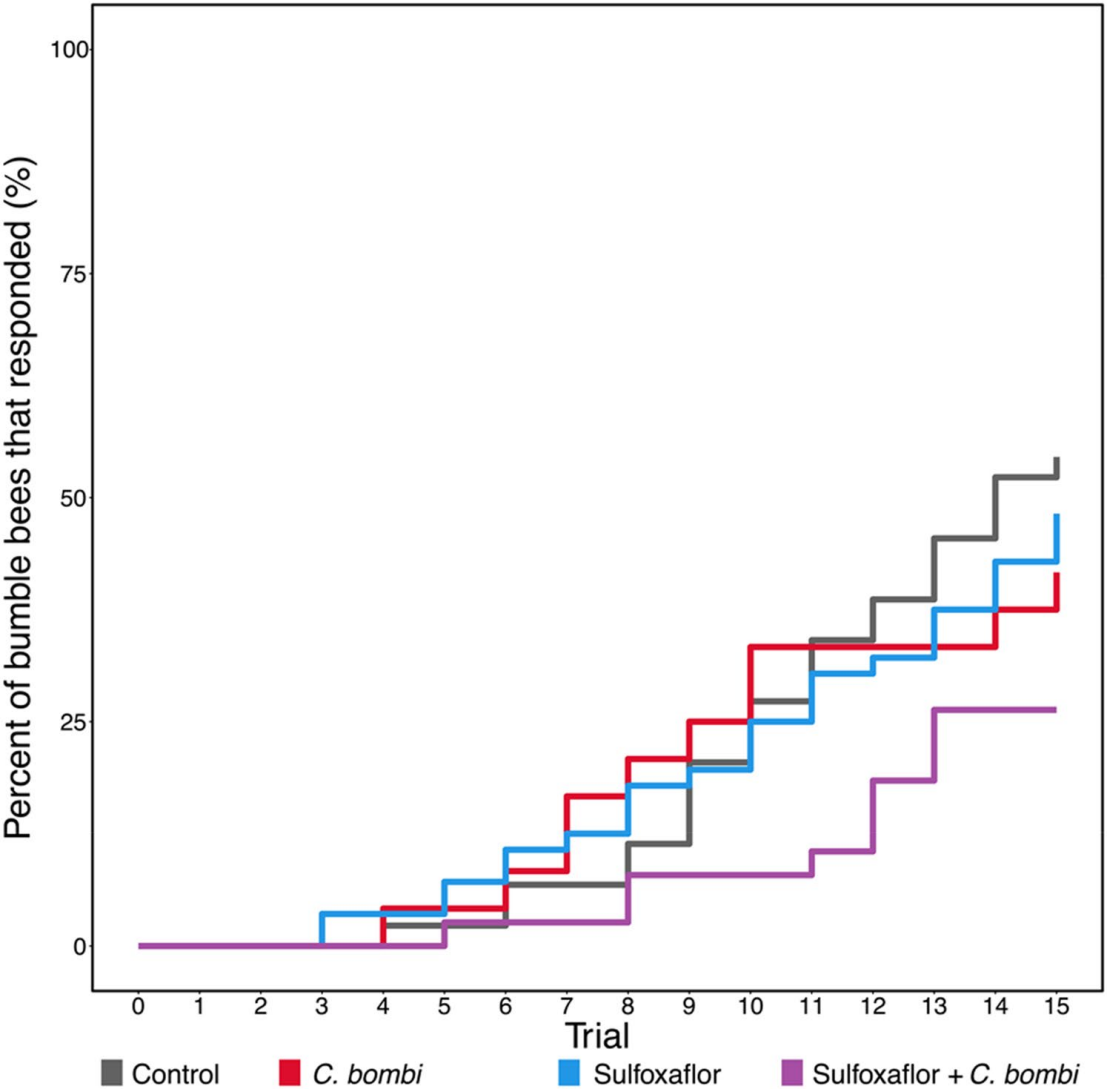
Learning Speed. Cumulative representation of the trials at which responsive workers showed their first conditioned response (control n = 44; sulfoxaflor n = 56; *C. bombi* n = 24; sulfoxaflor + *C. bombi* n = 38).

Finally, for Learning Speed (when a bee first showed a conditioned response), there was no evidence of significant impacts of exposure to sulfoxaflor, infection by *C. bombi* or their combination (Fig. 3; Cox Proportional Hazards sulfoxaflor PE =  −0.02, 95% CI [−1.15 to 1.11]; *C. bombi* PE = 0.00, 95% CI [−0.78 to 1.78]; sulfoxaflor and *C. bombi* PE = −0.33, 95% CI [−3.23 to 2.56]). Bees that had conditioned responses and were exposed to sulfoxaflor, *C. bombi* or were part of the control showed their first conditioned response, on average, on the 13th trial, whilst those exposed to both sulfoxaflor and *C. bombi* showed theirs on the 14th trial.

The size of individual bees did not have a significant effect on any of the three metrics: Responsiveness (GLM PE = 0.92, 95% CI [−0.62 to 2.45]), Learning Speed (Cox Proportional Hazards PE = 0.62, 95% CI [−0.41 to 1.66]). For Learning Level the best ‘standing model’ did not include the size term, and as such no results can be presented, nor can a size:treatment interaction results be presented.

For Responsiveness, we found no evidence of a significant interaction of size and treatment (GLM: control (PE) = 0.21, 95% CI [−0.89 to 1.32]; sulfoxaflor PE = 0.20, 95% CI [−0.83 to 1.24]; *C. bombi* PE = 0.19, 95% CI [−0.82 to 1.21]; sulfoxaflor and *C. bombi* PE = 0.18, 95% CI [−0.76 to 1.12]).

For Learning Speed, again we found no evidence of a significant interaction of size and treatment (Cox Mixed Effects Model (glm): control (PE) = 0.10, 95% CI [−0.52 to 0.72]; sulfoxaflor PE = 0.09, 95% CI [−0.55 to 0.73]; *C. bombi* PE = 0.08, 95% CI [−0.64 to 0.79]; sulfoxaflor and *C. bombi* PE = 0.12, 95% CI [−0.66 to 0.90]).

The colony of origin had no impact on any of the metrics (for further detail see the supplementary materials).

## Discussion

Using a modification of the classic PER learning paradigm, we found no evidence that exposure to sulfoxaflor, infection by *C. bombi,* or the combination of both stressors impact bumblebee olfactory learning. Previous research suggested that acute exposure to field realistic doses of sulfoxaflor does not impact olfactory learning in *B. terrestris* and *A. mellifera*^37^, and our findings are in line with these results for the former species and suggest that at the top end of non-lethal field realistic exposure there is still no impairment. Our findings also support the conclusion that *C. bombi*, whilst it impairs foraging and visual learning in bumblebees^34,52^, does not impact bumblebee olfactory learning^53^. While both stressors individually have not been found to impair bumblebee olfactory learning, it could not be discounted that their interaction might without verifying it, especially with a larger dose of sulfoxaflor than had previously been tested. By developing a new experimental paradigm for testing multiple stressors, that expands on existing protocols, we have demonstrated that, even together, these two stressors do not appear to impact bumblebee olfactory learning.

At 12 ng, the dose of sulfoxaflor that we used was 4.8 times greater than the largest dose used by Siviter et al. (2019) and almost 1/4 of the non-observable adverse effect level in bumblebees^54^. We chose such a high dose to simulate a worst-case scenario and to try and assess what quantity of sulfoxaflor might be required to observe an effect as a single stressor. Such a concentration is field realistic when strict guidelines are not followed during pesticide application, such as when farmers spray their crops too late, a factor that studies conducted for regulating bodies take into account^55,56^. The level of adherence to label restriction is not well documented, so whether this scenario is frequent is unknown. In a scenario where guidelines are properly followed, bumblebees would be unlikely to encounter such a dose. Therefore, if such an acute dose does not significantly affect bumblebee olfactory learning, it is unlikely that any sulfoxaflor application will. However, this does not address the potential effects of chronic exposure to sulfoxaflor, for which there is a lack of data, nor any effects sulfoxaflor may have on other forms of learning, such as visual learning.

The lack of evidence for sulfoxaflor affecting bumblebee learning is surprising in light of our knowledge of neonicotinoids. Neonicotinoid insecticides and sulfoxaflor are systemic insecticides and acetylcholine receptor competitive modulators^46,57^. Despite their similarities, it appears that these insecticides differ in their impact on bumblebee learning. Research conducted on the effects of neonicotinoids on pollinators has shown them to be detrimental to honeybee and bumblebee olfactory learning^43,58^. Currently, little is known on the biochemical reasons for this difference. There are differences in sulfoxaflor’s interaction with nAchRs, metabolic enzymes and resistance factors in insects compared to neonicotinoids^63^. However, the research that has been conducted so far has been in pest species such as aphids, fruit flies, whiteflies, leafhoppers and planthoppers^63^. The biochemical reasons behind the difference in the effects on learning of neonicotinoids and sulfoxaflor should be identified to help in the development of novel pesticides that could be less harmful to pollinators.

Regarding the effects of pathogens, as an activated immune system can have a negative impact on learning in honeybees and bumblebees^59–61^, the potential effects of pathogens should be studied. One possible candidate for such research is the microsporidian parasite of bumblebees *Vairimorpha bombi* (previously known as *Nosema bombi*) which, unlike *C. bombi*, can be present throughout the host’s body, including the brain^62^. *V. bombi* is already known to act additively with sulfoxaflor on the mortality of *B. terrestris* larvae^63^. There are limited data on how *V. bombi* might affect bumblebee learning, and there are conflicting data on how other members of the genus, *V. apis* and *V. ceranae*, might affect olfactory learning in honeybees^64–66^. However, in a recent study on the combined effects of the butenolide insecticide flupyradifurone, which is a nAChR inhibitor like sulfoxaflor, and *V. ceranae* on honeybee olfactory learning, Bell et al. (2020) found no significant interaction between the stressors, and olfactory learning was not significantly affected^67^.

Given the lack of predictive models for combined effects of stressors, and the particular importance of investigating interactions in otherwise low impact stressors, we deliberately chose two stressors where individual effects on PER were known. At least one of these, *C. bombi*, is known to exert significant impacts on bumblebee health when combined with nutritional stress^68^, as well as reducing mother queen survival and colony investment in producing sexual offspring when combined with exposure to neonicotinoids^23^, suggesting that there was potential for significant interaction effects between this and our second stressor, sulfoxaflor, which has a range of known impacts on bumblebees^63,69^. While our results for individual effects match those from previous studies^37,53^, giving us additional confidence in our finding that there is no combined impact of these stressors, we also acknowledge that reduced sample sizes in some analyses, may have resulted in a false negative result, particularly when considering the conservative nature of the tests we ran, such as binomial glm, but cannot determine if this was the case. Specifically, while our initial sample sizes for each treatment were similar to those of previous studies on the effects of pesticides on bumblebee learning^37,58^, it is possible our design lacked the power or sensitivity to detect the effect of sulfoxaflor + *C. bombi* exposure on responsiveness, which showed a 30% drop relative to the control. Because unresponsive bees were excluded from the Learning Level metric (to avoid duplicate testing) and because sulfoxaflor + *C. bombi* bees were comparably very unresponsive, the sample size for Learning Level was severely reduced, making a false negative result a real possibility. Future studies should build on the results of this study by including larger initial sample sizes to avoid the impacts of such dropout. Whilst it is not impossible that noise could have contributed to the lack of significant effects, we believe that it is unlikely. An example of a source of noise could have been bee age, which was not controlled for, but because our animals were selected haphazardly with respect to age, we do not believe it to be a reason for a by-treatment effect. Our selection process of using foragers, who are more mature, will also have reduced the uncontrolled variation.

Previous studies suggest that larger workers learn tasks more rapidly^70^ and have higher success levels in olfactory learning paradigms^58,71^. Larger workers have larger brains, and though their mushroom body lobes are relatively smaller than those of smaller workers, this is not the case for their calyx, which is an important structure for sensory input^72^. This means that larger bees have a larger calyx-to-mushroom body lobe ratio, enabling them to integrate more sensory information^72^. However, in contrast to expectations driven by these patterns, we did not find a significant effect of bee size in the PER paradigm. We have no explanation for this lack of an effect in this study.

Whilst our work highlights a lack of combined effects of sulfoxaflor and *C. bombi* on bumblebee olfactory learning, many questions have yet to be addressed. Since we used acute sulfoxaflor exposure, the potential effects of chronic exposure alone or combined with a pathogen remain unknown. Olfaction is not the only sense bumblebee workers make use of in foraging, so other forms of learning, such as visual learning, could also be examined. Whilst it is known that *C. bombi* impairs bumblebee visual learning^52^, it is unknown what effect sulfoxaflor might have, as is the case for a combination of the two. Our experiment limited itself to learning, so does not address questions pertaining to memory. Research should also be conducted to explore any combined effect of other pathogens and novel pesticides on pollinator fitness. *B. terrestris*, although less studied than *A. mellifera*, is still subject to more research than the vast array of wild bee species, and the effects of some pesticides vary between species of the same genus^73,74^. More research of this nature should be conducted on wild bees, as the results we obtained for *B. terrestris* may not be reflective of all bumblebees, not only because it is not a declining species, but also because results from pesticide effects on its fitness have differed from those on other species^9^.

## Methods

### Set-up of experimental colonies

Three colonies were bought from Agralan Ltd, UK. 10 workers from each colony were screened microscopically for parasites following Rutrecht & Brown (2009)^75^, and no parasites were found. Each colony was then evenly divided into two queenless sub-colonies to produce six sub-colonies. Prior to any further experimentation, the two sub-colonies from colony A had 125 and 128 individuals, this difference being due to some bee deaths. Those from colony B had 109 individuals and those from colony C had 110 each. The total number of workers was therefore 253 for colony A, 218 for colony B and 220 for colony C. Each sub-colony was placed into a colony box (27 × 20x13 cm) connected by a transparent plastic tube (25 cm long, 1 cm in diameter) to another empty colony box which served as a foraging arena containing an ad libitum supply of 50% w/w sucrose solution and pollen (Agralan Ltd, UK). The boxes containing the sub-colonies were covered to keep them in darkness, while the foraging arenas were left uncovered. These were setup in a room with natural light, to facilitate the development of normal foraging behaviour. This sub-colony design controls for strong colony effects in this host-parasite system (Schmid-Hempel et al. 1999)^76^ and whilst nest workers from queenless colonies perform better in olfactory learning tasks than those from queenright colonies (Evans et al., 2016)^77^, this factor was shared by all the sub-colonies.

### Parasite inoculation

Three days after splitting the colonies, one sub-colony from each of the three pairs was inoculated with *C. bombi*. *C. bombi* inoculum was produced from the purified faeces of 75 individuals from two infected lab colonies, following Cole (1970)^78^. The original source of the *C. bombi* strain was the faeces of wild *B. terrestris* queens captured that spring in Windsor Great Park, UK. Inoculum was diluted in 4 ml of 50% w/w sucrose solution for each of the three sub-colonies to drink from. The number of *C. bombi* cells provided corresponded to the number of individuals in each sub-colony multiplied by 10,000, for an average dose of 10,000 *C. bombi* cells per bee^53,79–81^.

With an inoculum concentration of 76,350 cells/µl, 16.37 µl of inoculum was presented to the 125 bees of one queenless split colony from colony A, 14.27 µl to the 109 bees of one queenless split colony from colony B and 14.40 µl to the 110 bees of one queenless split colony from colony C. Control sub-colonies were given 4 ml of 50% w/w sucrose solution without any parasite. After 24 h the inocula had been consumed and the sub-colonies were returned to ad libitum sucrose. All sub-colonies were screened at 7 and 10 days post-inoculation to confirm infection, and its absence in the control sub-colonies. This was done by looking at the faeces of ten workers from each sub-colony under the microscope. Bees that later underwent PER were then frozen at -80 °C and the 162 bees that were part of the final sample were dissected and their hindgut screened for presence or absence of *C. bombi*. All 100 bees from the uninfected treatments tested negative for *C. bombi* and all 62 bees from the infected treatments tested positive. Parasite load of individual bees was not recorded or considered in the analysis as Martin et al. (2018) found no relationship between parasite intensity and bumblebee olfactory learning^53^.

### Harnessing for PER

Over the course of 14 days, the sub-colonies were placed daily under red light and the five foragers that were first to present themselves at the feeder were collected from each sub-colony for harnessing (30 bees in total). Once harnessed, bees were held horizontally with modelling clay and prompted to extend their proboscis by touching their antennae with a droplet of 50% w/w sucrose solution. Bees that extended their proboscis were fed 50% w/w sucrose solution until satiated. Bees that did not feed were not used for PER. To avoid dehydration, the bees had damp paper roll put in their harnesses. They were then all left to starve together overnight for 18 h on a tray in a dark room at 24 °C, because leaving bumblebees to starve for 18 h is necessary to increase their responsiveness^72^. For additional details on harnessing and PER methodology on bumblebees, see Siviter et al.^37^.

### Sulfoxaflor exposure

Our stock solution of sulfoxaflor was prepared by diluting a powdered sulfoxaflor solution (Greyhound Chromatography and Allied Chemicals) in distilled water to a concentration of 400 mg/L, which was done with a magnetic stirrer for approximately 1 h whilst protecting it from light. It was then immediately stored in 1.5 ml aliquots at −20 °C. This stock solution was used in another study where it was verified by chemical testing^56^. The exposure solution was 12 ng of sulfoxaflor in 10 µl of a 25% w/w sucrose solution, prepared immediately before exposure, and fed to bees in the sulfoxaflor treatment which were prompted to feed and extend their proboscis by touching their antennae with a droplet of 50% w/w sucrose solution. Control bees were fed 10 µl of 25% w/w sucrose solution in the same manner as those exposed to sulfoxaflor. Bees that did not drink the whole treatment droplet were excluded from the experiment. PER was conducted 30 min after the last bee drank its given solution.

Since Siviter et al.^37^ did not find any effect for low field realistic doses of sulfoxaflor on bumblebee olfactory learning, we chose to test a worst-case scenario whilst limiting our experimentation to a sublethal dose. As such, our dose was almost four times lower than the non-observable adverse effect level in bumblebees of 44 ng^54^ and almost five times higher than the highest dose used by Siviter et al. (2019)^37^. We chose a dose of 12 ng as a range finder test we performed resulted in some mortality at higher doses. This dose was still conservatively low as field exposure studies performed for regulatory testing found that when strict mitigation methods for sulfoxaflor application are not applied, nectar concentrations could reach lethal levels^55,56^. Such worse case scenarios arise when farmers spray their crops when flower buds are present or already in bloom, which is too late to avoid pollinator contact with high doses of the insecticide.

### PER

We used Proboscis Extension Reflex protocol using lavender scent as a conditioned stimulus and a 50% w/w sucrose solution as an unconditioned stimulus. 4 µl of lavender oil (Calmer Solutions Ltd) was pipetted onto a strip of filter paper that was replaced every 24 trials. A stream of unscented air was constantly blown through a different tube to avoid bees misidentifying a change in air flow as a stimulus. Trials were conducted in a clear box connected to a vent duct hose that ensured air circulation. During trials, bees were placed 3 cm from the odour tube. They were exposed to unscented air for 5 s, then to scented air for 10 s. 6 s after the start of the scented airflow the antennae were stimulated with 0.8 µl sucrose solution from a pipette, and an unconditioned response was recorded if a bee extended its proboscis. A conditioned response was recorded if a bee extended its proboscis during the first 6 s of scented airflow, thereby demonstrating learning in the form of classical conditioning. Bees that showed either response were fed the 0.8 µl sucrose solution as a reward to reinforce the behaviour. Each bee underwent 15 trials, and 3 unscented probe trials that were randomly placed between the 1st and 5th, 5th and 10th and the 10th and 15th scented trials. The purpose of these probes was to ensure that bees would be developing a conditioned stimulus solely in response to the lavender scent. Bees that would have extended their proboscis during a probe trial would not have been included in the analysis, but none did. Therefore, each bee underwent a total of 18 trials with an interval of approximately 12 min between each one.

Bees that did not extend their proboscis in at least 5 trials when their antennae were stimulated with sucrose solution were excluded from the analysis. Bees that died whilst undergoing PER were also excluded from the analysis but recorded as deaths.

Tested bees were then frozen at −80 °C. Three intertegular measurements were taken with a Mitutoyo digital calliper to obtain mean intertegular values, since the absorption rate of the insecticide and its effects on bee cognition may be affected by a bee’s size^82,83^.

A total of 326 bumblebees underwent PER (81 control, 81 sulfoxaflor, 82 *C. bombi*, and 82 sulfoxaflor + *C. bombi*). Of these, 161 bees did not extend their proboscis in at least 5 trials when their antennae were stimulated with sucrose solution and so were excluded from the analysis (35 control, 25 sulfoxaflor, 58 *C. bombi*, and 43 sulfoxaflor + *C. bombi*). This statistically differs by treatment (Chi-Squared, X^2^ = 27.3, df = 3, *p* < 0.0001), with *C. bombi* having the highest exclusion rate. This could be driven by impacts of the parasite on host activity or sensitivity. 3 bees died during the trials, 2 of the control treatment and one of the sulfoxaflor + *C. bombi* treatment. Overall, 164 bees were excluded from the statistical analysis, for a final sample size of 162 bumblebees (44 control, 56 sulfoxaflor, 24 *C. bombi*, and 38 sulfoxaflor + *C. bombi*).

### Statistical analysis

R programming software version 4.0.5^84^ was used for statistical analysis, along with the packages survminer version 0.4.8^85^, survival version 3.1–12^86^ and MuMIn version 1.43.17^87^. Graphs were made in R using ggplot2 version 3.3.3^88^.

Three dependent variables were analysed: Responsiveness (if a bee displayed at least one conditioned response to the stimulus), Learning Level (the number of conditioned responses for bees that displayed at least one conditioned response) and Learning Speed (the trial in which a bee displayed its first conditioned response). We did not use a mixed effects model as there were fewer than 5 levels for Colony of Origin.

For Responsiveness and Learning Speed, models with different covariates were compared to each other using their AICc values to see which one was best supported. If none had ≥ 95% AICc support, we added the next best supported model until ≥ 95% cumulative support was met^37,89^. The final model set was averaged, meaning that it incorporated several models. Parameter estimates (PE) and 95% confidence intervals (CI) are reported.

A Generalised Linear Effects Model (GLM) with binomial distribution was run for Responsiveness, the full model being (Responsiveness ~ Treatment + Colony of Origin + Size + Treatment:Colony of Origin + Treatment:Size + Colony of Origin:Size + Treatment:Colony of Origin:Size). However, the full model itself caused overfitting issue, so it was dropped, but the remaining models were appropriately fitted.

For Learning Level a GLM using a Poisson regression caused model fitting issues, with evidence of overdispersion. A quasi-Poisson model was used instead, with pairwise model selection using a Chi-Squared test as a quasi-Poisson model does not yield an AIC since it is not resolved via full maximum likelihood. Models were compared in pairs, with the best supported model being retained as the standing ‘best model’. The least complex model was retained as the standing ‘best model’ unless the more complex model was a significantly better fit α = 0.05.

A GLM with quasi-poisson error structures was run for Learning Level, the full model being (Learning Level ~ Treatment + Colony of Origin + Size + Treatment:Colony of Origin + Treatment:Size + Colony of Origin:Size + Treatment:Colony of Origin:Size). Bees that did not extend their proboscis at all were excluded from this analysis, as not doing so would have caused a duplication of the Responsiveness test.

A Cox Proportional-Hazards Model was run for Learning Speed, with the full model being (Learning Speed ~ Treatment + Size + Treatment:Size + Colony of Origin). However, the full model itself caused overfitting, so it was dropped, but the remaining models were appropriately fitted.

## Supplementary Information

Supplementary Information.

## Data Availability

We intend to archive our data with Data Dryad.
