# Supplementary material for: No effect of dual exposure to sulfoxaflor and a trypanosome parasite on bumblebee olfactory learning
Source: Sci Rep. 2022 May 21;12:8611. doi: 10.1038/s41598-022-12714-3 (PMC9124203; doi:10.1038/s41598-022-12714-3)
Supplement: Supplementary file 1 — Supplementary Information. [file 41598_2022_12714_MOESM1_ESM.docx]

**No effect of dual exposure to sulfoxaflor and a trypanosome parasite on bumblebee olfactory learning.**

Owen P. Vaughan; Edward A. Straw; Alberto Linguadoca; Mark J. F. Brown

**Supplementary Figure 1.** Responsiveness: Model selection and AIC results using the R programming software package MuMIn version 1.43.17 (Bartoń, 2020)

| Model name | Predictors | AIC | ΔAIC from best model | AIC weight | Included in the final model |
| --- | --- | --- | --- | --- | --- |
| Mnul | 1+ Colony_origin | 223.5 | 7.93 | 0.004 | No |
| M1 | Colony_origin*Treatment*size | NA | NA | NA | No |
| M2 | Colony_origin*Treatment | 232.1 | 16.52 | 0.000 | No |
| M3 | Treatment*size | 219.3 | 3.80 | 0.028 | No |
| M4 | Colony_origin*size | 217.2 | 1.71 | 0.081 | Yes |
| M5 | Colony_origin:Treatment | 232.1 | 16.52 | 0.000 | No |
| M6 | Treatment:size | 216.2 | 0.68 | 0.135 | Yes |
| M7 | Colony_origin:size | 215.8 | 0.25 | 0.167 | Yes |
| M8 | Treatment+size | 215.8 | 0.31 | 0.162 | Yes |
| M9 | Colony_origin+size | 216.1 | 0.61 | 0.140 | Yes |
| M10 | Colony_origin+Treatment | 222.6 | 7.09 | 0.005 | No |
| M11 | Colony_origin+Treatment+size | 217.3 | 1.75 | 0.079 | Yes |
| M13 | Treatment | 222.9 | 7.32 | 0.005 | No |
| M14 | size | 215.5 | 0.00 | 0.190 | Yes |

**Supplementary Figure 2.** Learning Level: Model selection and results

| Model name | Predictors | Best model | Residual deviance | Significance of the difference compared to the null model (*p* =) |
| --- | --- | --- | --- | --- |
| qMnull | 1 | Yes | 119.61 | *na* |
| qM1l | Treatment | No | 113.50 | 0.3605 |
| qM2l | Treatment+size | No | 107.45 | 0.1387 |
| qM3l | Treatment+size+Colony_origin | No | 105.25 | 0.2232 |
| qM4l | Treatment+Colony_origin | No | 110.08 | 0.4023 |
| qM5l | size | No | 114.47 | 0.09007 |
| qM6l | Colony_origin | No | 114.72 | 0.2713 |
| qM7l | Treatment*size | No | 106.95 | 0.4277 |
| qM8l | Treatment*Colony_origin | No | 104.26 | 0.7383 |
| qM9l | Colony_origin*size | No | 110.19 | 0.3973 |
| qM10l | Treatment*Colony_origin*size | No | 91.56 | 0.8854 |

**Supplementary Figure 3.** Learning Speed: Model selection and AIC results

| Model name | Predictors | AIC | ΔAIC from best model | AIC weight | Included in the final model |
| --- | --- | --- | --- | --- | --- |
| MnulS | 1 | 686.0 | 7.16 | 0.007 | No |
| M1S | Colony_origin*Treatment*size | NA | NA | NA | No |
| M2S | Colony_origin*Treatment | 696.7 | 17.84 | 0.000 | No |
| M3S | Treatment*size | 683.6 | 4.79 | 0.021 | Yes |
| M4S | Colony_origin*size | 681.9 | 3.03 | 0.052 | Yes |
| M5S | Colony_origin:Treatment | 696.7 | 17.84 | 0.000 | No |
| M6S | Treatment:size | 680.5 | 1.72 | 0.099 | Yes |
| M7S | Colony_origin:size | 679.2 | 0.33 | 0.199 | Yes |
| M8S | Treatment+size | 680.1 | 1.24 | 0.126 | Yes |
| M9S | Colony_origin+size | 679.4 | 0.57 | 0.177 | Yes |
| M10S | Colony_origin+Treatment | 685.0 | 6.20 | 0.011 | No |
| M11S | Colony_origin+Treatment+size | 681.8 | 2.96 | 0.054 | Yes |
| M12S | Colony_origin | 685.1 | 6.33 | 0.010 | No |
| M13S | Treatment | 685.1 | 6.26 | 0.010 | No |
| M14S | size | 678.8 | 0.00 | 0.235 | Yes |

**Colony of origin:**

The colonies of origin did not have significant effects on any of the metrics for which colony of origin was included in the chosen model. Colony A was used as a control.

For Responsiveness: General Linear Model (GLM), Colony B (PE) = -0.61, 95% CI [-5.86 to 4.62], Colony C PE = -0.19, 95% CI [-3.78 to 3.39].

For Learning Speed: Cox Proportional Hazards, Colony B (PE) = -0.18, 95% CI [-2.55 to 2.18], Colony C PE = -0.19, 95% CI [-2.75 to 2.37].

**Additional information on bee numbers:**

In total, over the course of the experiment, 420 bees were harnessed to account for overnight deaths (n=25), meaning that 140 bees of each of the three colonies, or 105 bees of each of the four treatment groups were harnessed. Of the 30 bees harnessed daily, 24 were expected to undergo PER for a total of 336. However, trial 12 was run with only 17 bees because 3 bees did not consume any sucrose solution prior to PER and there were 10 overnight deaths. Trial 13 was therefore run with 21 bees to try and ensure that an equal number of bees of each treatment had undergone PER.

A total of 326 bumblebees underwent PER: 81 control (27 from colony A and C, 25 from B), 81 sulfoxaflor (27 from each colony), 82 *C. bombi* (27 from colony A and B, 28 from C) and 82 sulfoxaflor and *C. bombi* (27 from colony A and B, 28 from C).

161 bees that did not extend their proboscis in at least 5 trials when their antennae were stimulated with sucrose solution were excluded from the analysis: 35 control (7 from colony A, 7 from B and 21 from C), 25 Sulfoxaflor (4 from colony A, 7 from B and 14 from C), 58 *C. bombi* (13 from colony A, 23 from colony B and 22 from C) and 43 sulfoxaflor and *C. bombi* (9 from colony A, 15 from B and 19 from C). 3 bees died during the trials, 2 of the control treatment both being from colony B and one of the sulfoxaflor and *C. bombi* treatment being from colony C, and were therefore also excluded. A total of 164 were excluded from the analysis.

The final sample size was 162 bumblebees: 44 control (20 from colony A, 18 from B and 6 from C), 56 Sulfoxaflor (23 from colony A, 20 from B and 13 from C), 24 *C. bombi* (14 from colony A, 4 from B and 6 from C) and 38 sulfoxaflor and *C. bombi* (18 form colony A, 12 from B and 8 from C).

**Dose determination:**

Our acute dose of 12ng/bee was within the distribution of daily doses calculated from EFSA 2019 and EPA 2019 (Fig. 4). Concentrations in nectar were extracted from individual experiments included in the two regulatory reports and collated into a common dataset. Concentrations below the limits of quantification (0.01 mg/kg) and detection (0.003 mg/kg) were considered to be half of the respective values. All concentrations of sulfoxaflor in nectar were converted into daily pesticide intakes by assuming a sugar consumption of 73 mg/bee/day (EFSA, 2013) and a sugar concentration in nectar of 30%, as used by EPA 2014 in the exposure assessment. This results in a daily consumption of contaminated nectar of 243 mg/bee/day, which is a best-case compared to EFSA 2013, which assumes up to 149 mg sugar/bee/day and a sugar concentration in nectar of 15%. Daily pesticide intake was plotted against our acute dose to visually assess the field realism of our dosage. When our doses were plotted against the estimated pesticide intake from individual crops (Fig. 5), results show that our dosage was largely conservative, as it was representative of the lower end of the distribution of estimated daily intake of sulfoxaflor in each crop. There were two exceptions, which were oilseed rape and pumpkin from the EPA dataset. However, for both crops, when the EFSA dataset was considered, our exposure was still representative of a best-case scenario.

Both datasets result from controlled experiments where sulfoxaflor was sprayed at label rates during or immediately prior to flowering. Upon flowering, nectar samples were collected and analysed from flowers or foraging bees for up to 21 days. Since the crops were mostly sprayed while in bloom, the dataset represents a realistic worst-case exposure scenario. However, exposure from pollen, for which concentrations were higher was not considered in our assessment. Therefore, our estimate still shows that 12ng is a conservative acute exposure. It was necessary for us to select a non-lethal dose. Therefore, our dose needed to be at the lower end of the distribution in Fig. 5.

**Supplementary Figure 4.** Distribution of estimated daily intake of sulfoxaflor calculated from EFSA 2019 and EPA 2019 papers. The dashed line represents 12 ng/bee/day of sulfoxaflor. Outliers have been cropped out to aid in visualisation.


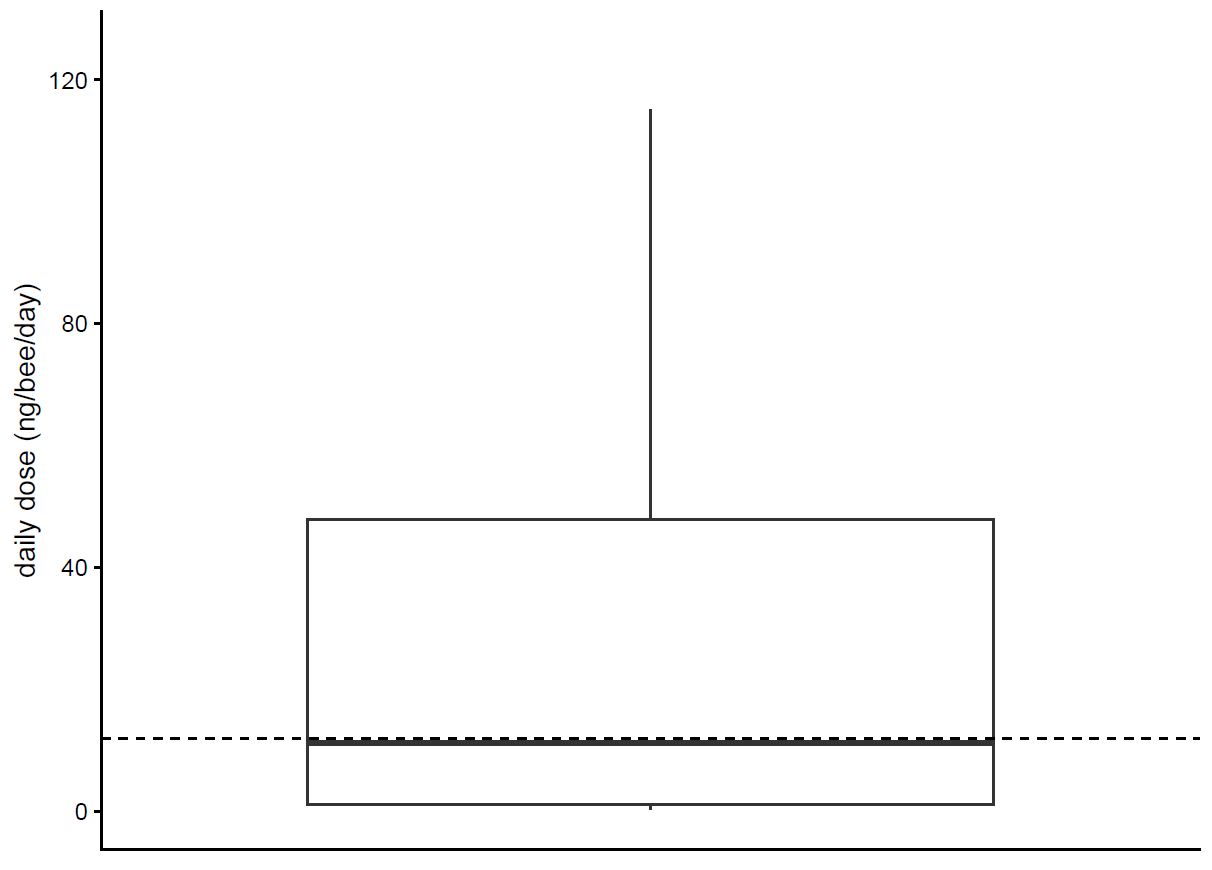


**Supplementary Figure 5.** Distribution of estimated daily intake of sulfoxaflor calculated from EFSA 2019 and EPA 2019 papers aggregated by source and crop. Different colours of the jittered raw data points represent different trials. The dashed line represents 12 ng/bee/day of sulfoxaflor.


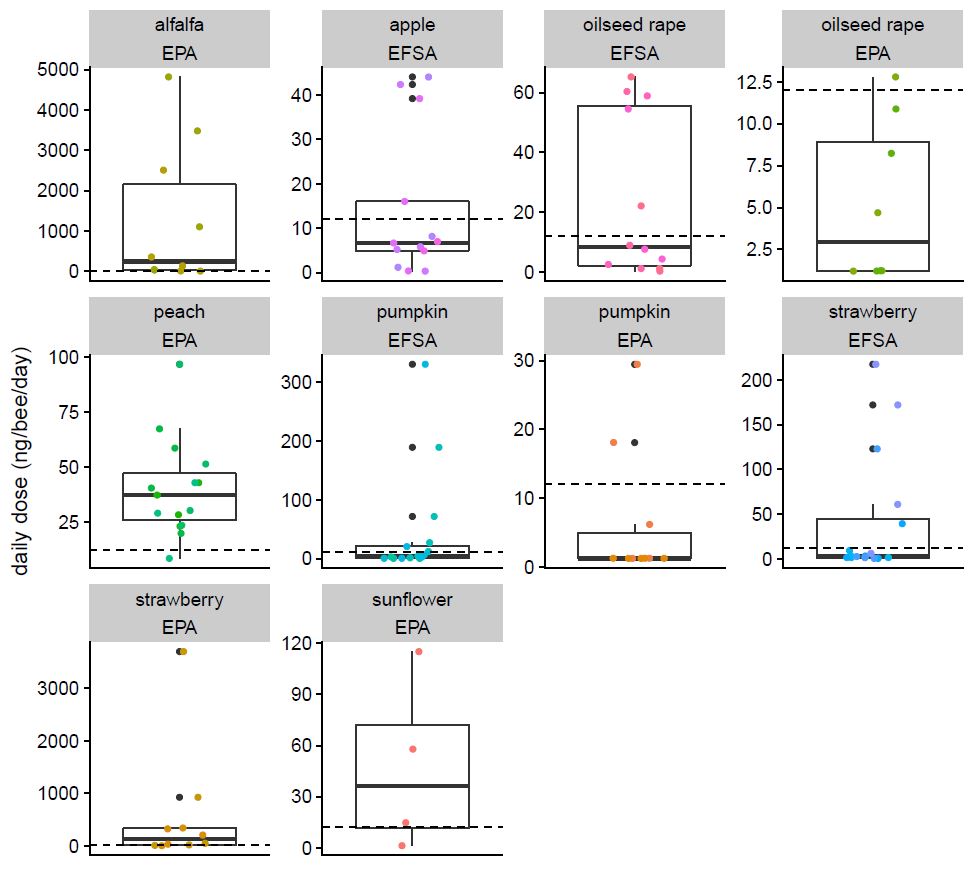


References:

Bartoń, K. (2020). MuMIn: Multi-Model Inference. R package ver. 1.43. 17. *CRAN: The Comprehensive R Archive Network*, Berkeley, CA, USA.

EFSA, P. P. R. (2013). Panel (EFSA Panel on Plant Protection Products and their Residues), 2013. *Guidance on tiered risk assessment for plant protection products for aquatic organisms in edge‐of‐field surface waters. EFSA Journal*, *11*(7), 3290. doi:10.2903/j.efsa.2013.3295

EPA. (2014). Guidance for Assessing Pesticide Risks to Bees. <https://www.epa.gov/sites/default/files/2014-06/documents/pollinator_risk_assessment_guidance_06_19_14.pdf>

EFSA., Abdourahime, H., Arena, M., Auteri, D., Barmaz, S., Ctverackova, L., De Lentdecker, C., Ippolito, A., Kardassi, D., Messinetti, S., Molnar, T., Saari, K, E., Sharp, R., Streissl, F., Sturma, J., Szentes, C., Tiramani, M., Vagenende, B., Van Dijk, J., & Villamar‐Bouza, L. (2019). Peer review of the pesticide risk assessment for the active substance sulfoxaflor in light of confirmatory data submitted. *EFSA Journal*, *17*(3). doi:10.2903/j.efsa.2019.5633

EPA. (2019). Decision memorandum supporting the registration decision for new uses of the active ingredient sulfoxaflor on alfalfa, cacao, citrus, corn, cotton, cucurbits, grains, pineapple, sorghum, soybeans, strawberries and tree plantations and amendments to labels *Epa (2019)*, pp. 1-30
